# Exposure to excessive heat and impacts on labour productivity linked to cumulative CO_2_ emissions

**DOI:** 10.1038/s41598-019-50047-w

**Published:** 2019-09-23

**Authors:** Yann Chavaillaz, Philippe Roy, Antti-Ilari Partanen, Laurent Da Silva, Émilie Bresson, Nadine Mengis, Diane Chaumont, H. Damon Matthews

**Affiliations:** 1 Ouranos Inc, 550 rue Sherbrooke Ouest, Tour Ouest 19e étage, Montréal, QC H3A 1B9 Canada; 2 Department of Geography, Planning and Environment, Concordia University, 1455 boulevard de Maisonneuve Ouest, Montréal, QC H3G 1M8 Canada; 3 Finnish Meteorological Institute, Climate System Research, P.O. Box 503, 00101 Helsinki, Finland; 4 Université du Québec en Abitibi-Témiscamingue, 445 Boulevard de l’Université, Rouyn-Noranda, QC J9X 5E4 Canada; 5 Simon Fraser University, Department of Geography, 8888 University Drive, Burnaby, BC V5A 1S6 Canada; 6 Helmholtz Centre for Ocean Research Kiel (GEOMAR), Düsternbrooker Weg 20, Kiel, D-24105 Germany

**Keywords:** Climate and Earth system modelling, Environmental health, Projection and prediction

## Abstract

Cumulative CO_2_ emissions are a robust predictor of mean temperature increase. However, many societal impacts are driven by exposure to extreme weather conditions. Here, we show that cumulative emissions can be robustly linked to regional changes of a heat exposure indicator, as well as the resulting socioeconomic impacts associated with labour productivity loss in vulnerable economic sectors. We estimate historical and future increases in heat exposure using simulations from eight Earth System Models. Both the global intensity and spatial pattern of heat exposure evolve linearly with cumulative emissions across scenarios (1% CO_2_, RCP4.5 and RCP8.5). The pattern of heat exposure at a given level of global temperature increase is strongly affected by non-CO_2_ forcing. Global non-CO_2_ greenhouse gas emissions amplify heat exposure, while high local emissions of aerosols could moderate exposure. Considering CO_2_ forcing only, we commit ourselves to an additional annual loss of labour productivity of about 2% of total GDP per unit of trillion tonne of carbon emitted. This loss doubles when adding non-CO_2_ forcing of the RCP8.5 scenario. This represents an additional economic loss of about 4,400 G$ every year (i.e. 0.59 $/tCO_2_), varying across countries with generally higher impact in lower-income countries.

## Introduction

Excessive heat exposure has many potential socioeconomic impacts, including health effects, loss of labour productivity, higher death rates and higher energy demand [e.g.^1^]. Previous analyses have developed relationships between heat exposure thresholds and health recommendations with respect to recommended rest time during labour^2–5^. In these studies, it was found that above a given level of heat exposure, workers need to increase the amount of rest during working hours by amounts ranging from 10 to 40 minutes per hour of active work^5^. Though workers from different economic sectors and countries are impacted differently, these health guidelines nevertheless indicate the potential for labour productivity loss with increasing exposure to extreme heat conditions. In 2017, observations show that 153 billion hours of labour were lost worldwide as a result of heat exposure, which represents an increase of 62 billion hours lost relative to the year 2000^6^. Given the importance of heat exposure as a driver of socioeconomic impacts, some recent analyses have suggested that maximum temperature values could scale linearly with cumulative CO_2_ emissions (CCE) [e.g.^7,8^]. Here, we assess to what extent cumulative CO_2_ emissions are linked to increased extreme heat exposure and resulting labour productivity loss across future climate change scenarios.

The Transient Climate Response to cumulative CO_2_ Emissions (TCRE) represents the linear response of global temperatures to CCE, considering physical climate processes and the dynamics of natural carbon sinks. The TCRE is approximately constant in time and independent of the emission pathway^8–18^, and has also been shown to be a reasonable predictor of the regional climate response to cumulative emissions^19,20^. Here, we begin by extending the generalized TCRE framework from annual and seasonal trends in regional climate to changes in heat exposure. We define heat exposure using thresholds of the Wet-Bulb Globe Temperature (WBGT) index, first developed in the mid-twentieth century^21^ and widely used for industry labor standard^22^.

We use here a simplified version of the WBGT, which is calculated using average solar irradiation and wind speeds. This version is easy to reproduce from climate model outputs^23^. From this indicator, we then define the annual total heat exposure as the integral of daily WBGT values in a year above six pre-defined thresholds, labelled as light, medium, strong, very strong, extreme and deadly (see Methods). Finally, we quantify the response of heat exposure to CCE in eight CMIP5 Earth system models (ESMs, i.e. models which include a dynamic carbon cycle^24^) across a range of emission pathways.

Following this estimate of the increase in heat exposure due to cumulative CO_2_ emissions, we combine our results with datasets provided by the International Labour Organization^25^ to estimate how labour productivity loss due to heat exposure can be linked to CCE. We express productivity loss as a relative extra annual loss of total GDP in addition to the annual loss that could already have been experienced during the pre-industrial period. This indicator is assessed for economic sectors that are considered as vulnerable to heat exposure: agriculture, mining and quarrying, manufacturing and construction sectors^26^. For future scenarios, we assume that socioeconomic conditions do not vary from current conditions (i.e. 2017) over the entire study period. This approach therefore allows us to quantify an upper bound of the increase in labour productivity loss that would occur in the absence of socioeconomic adaptation to changing climate conditions.

## Results

### Evolution of heat exposure

**Figure 1:**
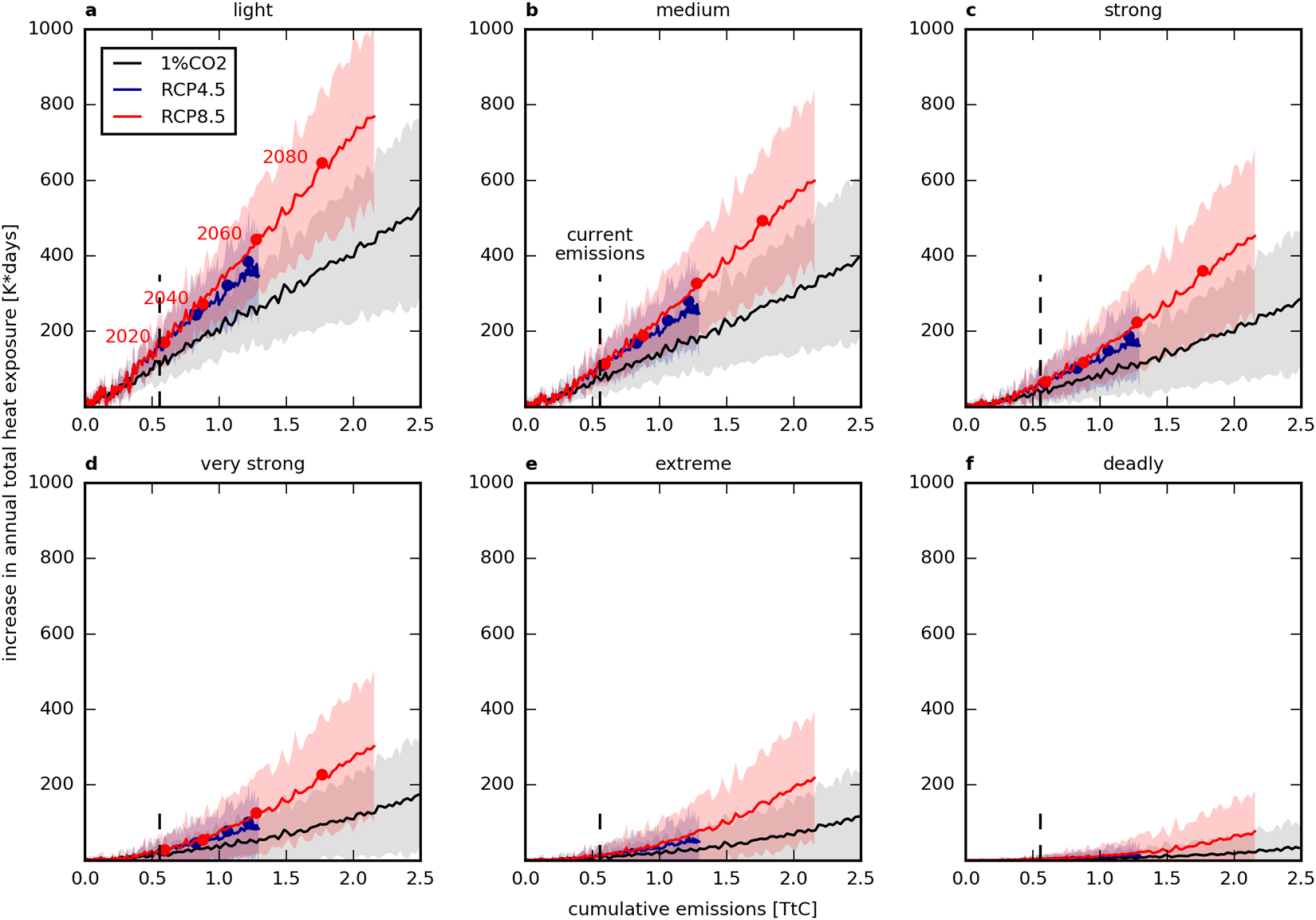
Global increase in heat exposure per carbon emission. Increase in the annual total heat exposure ${I}_{\Sigma }$ over land as a function of cumulative carbon emissions for the different emission scenarios (1% CO_2_, RCP4.5 and RCP8.5) compared to the pre-industrial period (1861–1880). Different WBGT thresholds are considered: light (**a**), medium (**b**), strong (**c**), very strong (**d**), extrem**e** (**e**) and deadly (**f**). The transient response to cumulative emissions is robustly linear from (**a**) to (**d**), while too few periods over 90 and 95 °F are simulated to build a significant correlation with emissions. Envelopes correspond to the *σ*-interval of the inter-model spread.

At a global scale, the annual total heat exposure over land areas increases linearly as a function of CCE for the four lowest WBGT thresholds selected. In the 1% CO_2_ (CO_2_-only) scenario, heat exposure above the light threshold increases by $213.1\pm 105.1\,{\rm{K}}$-days per trillion tonne of carbon (TtC) (see Fig. 1a–d and Supplementary Table S3). As current CCE are estimated at 555 PgC^27^, this represents an estimated increase in heat exposure of about $118.3\pm 58.3\,{\rm{K}}$-days relative to the beginning of the pre-industrial period. Heat exposure above the extreme and deadly thresholds also increases with cumulative emissions, by $55.30\pm 53.31$ and $18.44\pm 28.37\,{\rm{K}}$-days per TtC, respectively (see Fig. 1e,f); however, given the small signals and high inter-model spread, we were not able to demonstrate a statistically robust linear relationship with CCE. Across all WBGT thresholds, the RCP scenarios show a more rapid and more robust increase in heat exposure compared to the 1% CO_2_ scenario as a result of additional positive non-CO_2_ forcing.

**Figure 2:**
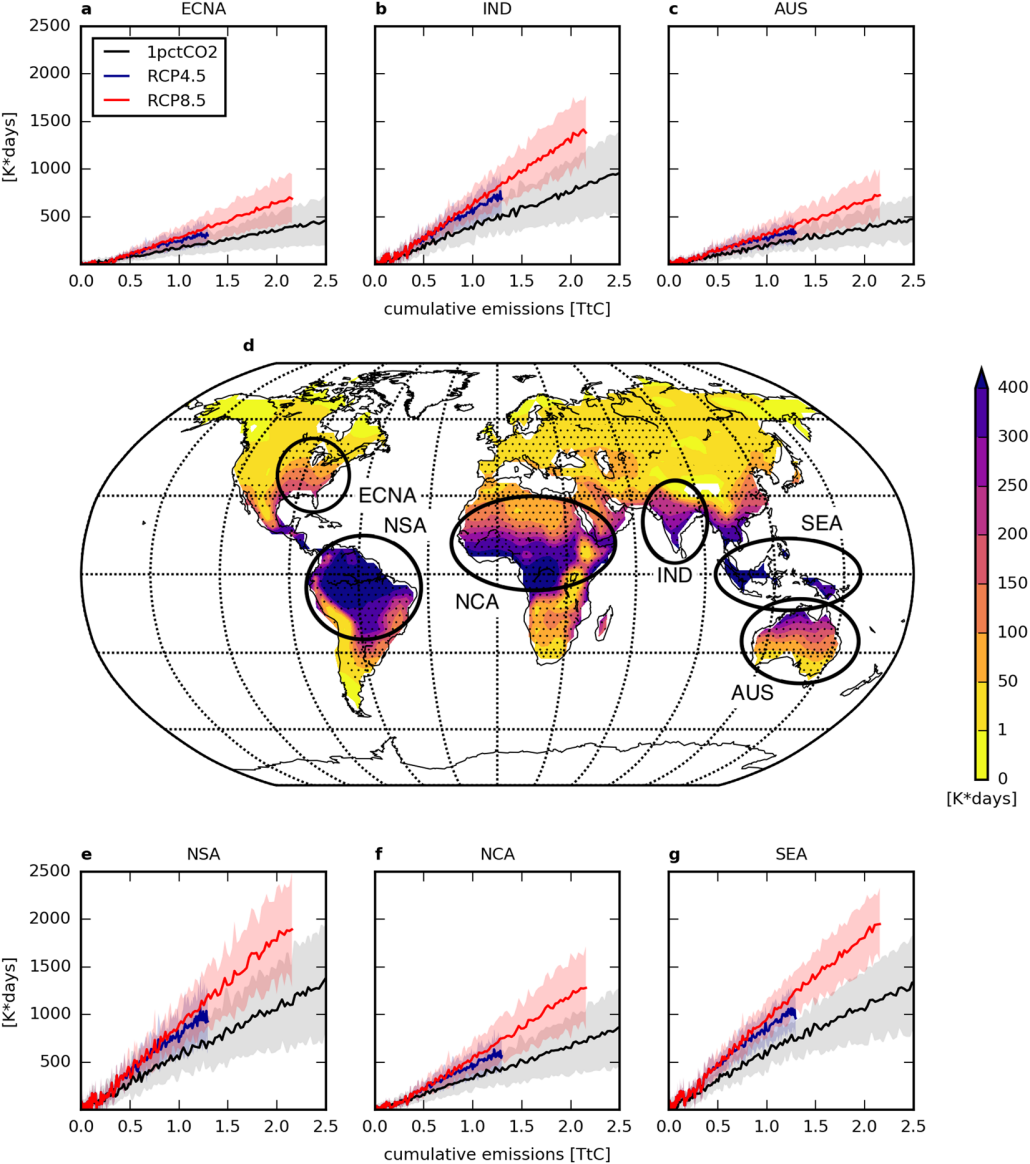
Spatial representation of the increase in heat exposure with a light WBGT threshold. (**d**) Regional increase in the annual total heat exposure ${I}_{\Sigma }$ in a 1.5 °C-warmer world compared to the pre-industrial period (1861–1880) according to the 1% CO_2_ scenario. Dotted regions correspond to where at least 75% of the models agree on the significance of the change. Six SREX regions (or aggregation of SREX regions) are highlighted for their significant change. (**a–c**,**e–g**) Regional increase in the annual total heat exposure ${I}_{\Sigma }$ linked to cumulative carbon emissions under three different scenarios (1% CO_2_, RCP4.5 and RCP8.5) in East-Central North America, India, Australia, Northern South America, Northern Central Africa and South-East Asia, respectively. Envelopes correspond to the *σ*-interval of the inter-model spread.

Regional heat exposure increases are larger in equatorial regions compared to subtropical and temperate regions (Fig. 2). Despite the polar amplification of temperature increase, lower temperatures in high-latitude regions and higher humidity in the tropics lead to larger increases in heat exposure in tropical regions. Due to the combination of high temperature and high humidity, some areas experience more than 300 K-days of increase in total heat exposure (above the light threshold) at the time that global temperature reaches 1.5 °C above pre-industrial levels (Fig. 2d). This highlights the unequal distribution of climate change impacts across the globe, as well as the potential for substantial regional heat exposure impacts in the near future. Figure 2 also shows the response of regional annual total heat exposure to CCE over six individually aggregated regions defined in the IPCC Special Report on Extreme Events (SREX, see Supplementary Table S2)^28^. In those regions with a sufficiently robust signal such as South-East Asia, India or the Eastern Central part of North America, the regional changes in heat exposure are significantly linear with respect to increasing CCE.

**Figure 3:**
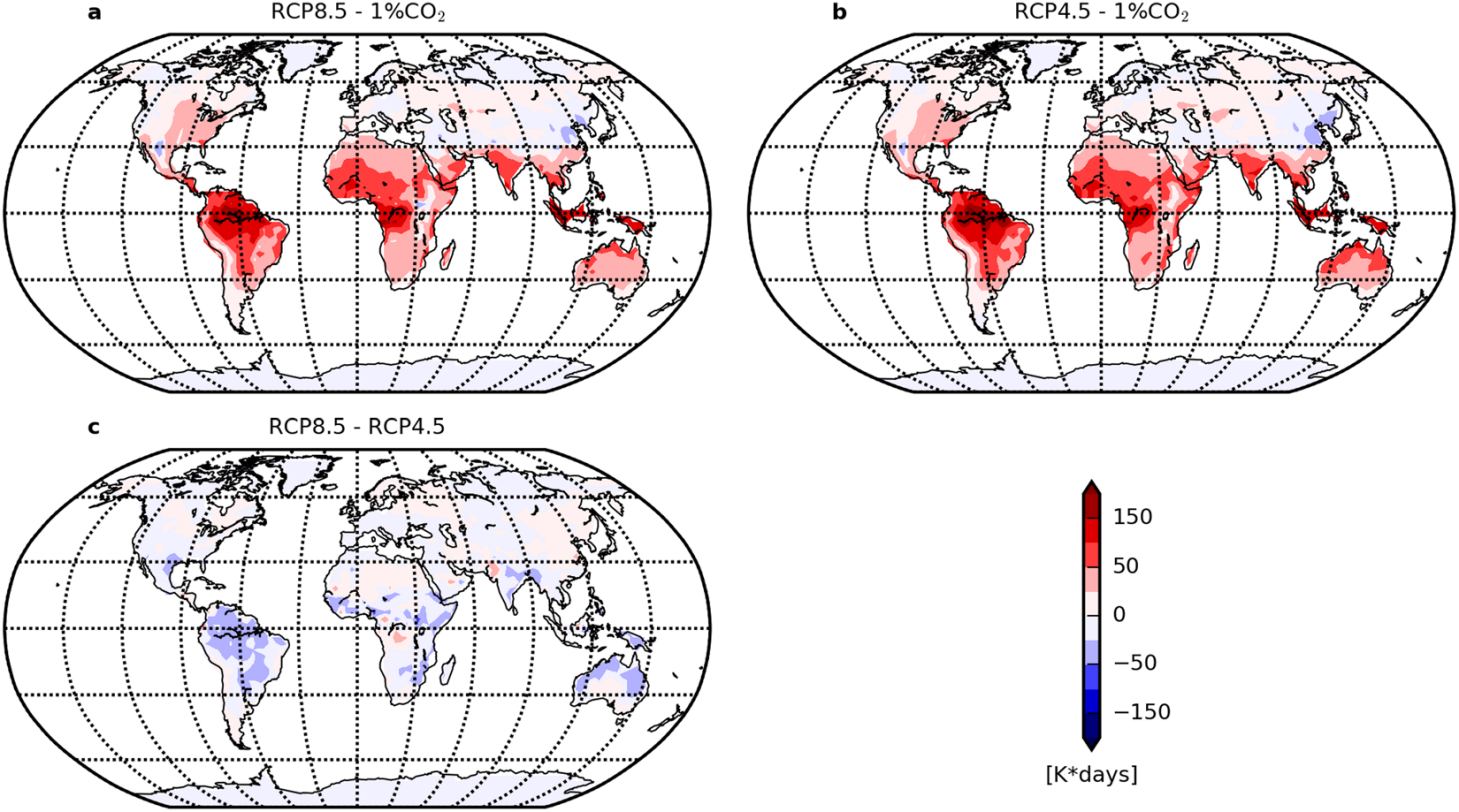
Comparison between different scenarios of the increase in heat exposure with a light WBGT threshold in a 1.5 °C-warmer world. (**a**–**c**) Differences in the increase in the annual total heat exposure ${I}_{\Sigma }$ between scenarios of emissions. RCP scenarios have a greater response due to non-CO_2_ forcing not implemented in the 1% CO_2_ scenario. RCP4.5 and RCP8.5 have a similar response.

The spatial pattern of the increase in annual total heat exposure remains robust across all scenarios (see Fig. 2d and Supplementary Fig. S2a,b). However, the three scenarios differ in the amplitude of changes: both RCP scenarios show an amplification of regional heat exposure in low latitudes for the same global temperature increase, compared to the 1% CO_2_ scenario (Fig. 3). On one hand, this pattern amplification occurs as a result of the positive non-CO_2_ greenhouse gas forcing, which tends to amplify heat exposure increases everywhere [consistent with Baker *et al*. (2018)^29^. On the other hand, emissions of short-lived aerosols (leading to negative non-CO_2_ forcing) might act to locally mitigate this increase. This second effect is notable in areas of high local aerosol emissions, such as the Chinese East Coast (see Fig. S3 in the Supporting Information^30^). These high aerosol emissions likely explain the slightly lower regional increases in heat exposure for RCP4.5 and RCP8.5 than for the 1% CO_2_ scenario, though analysis of consistent simulations with and without aerosol forcing would of course be required to confirm the magnitude of this effect.

Other metrics of heat exposure such as mean and maximum intensity and duration of heat periods also increase linearly as a function of CCE (see Supplementary Figs S5–S8). However, the increase in annual total number of days with heat exposure shows evidence of saturation at higher levels of CCE for the light to strong WBGT thresholds, reaching 50 to 100 additional days per year depending on the emission scenario (see Supplementary Fig. S9, consistent with findings of Dunne *et al*. (2013)^3^). In this case, regions that are strongly affected, such as Central Africa, South-East Asia and the Amazon region, begin to experience light to strong heat exposure during every day of the year. The first areas to be exposed to this phenomenon with the strong WBGT threshold appear from approximately 1.4 °C of global temperature increase compared to the pre-industrial period, depending on the ESM selected.

### Labour productivity loss

**Figure 4:**
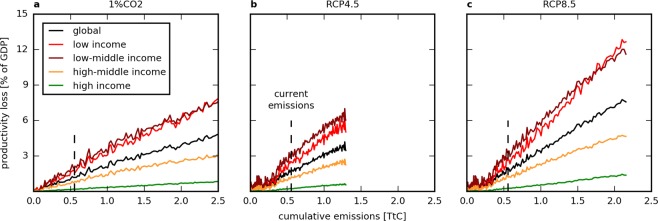
Annual labour productivity loss due to the increase in heat exposure classified by income class based on GDP per capita. Evolution of ${L}_{\Sigma }$ as a function of cumulative carbon emissions for the 1% CO_2_ (**a**), RCP4.5 (**b**) and RCP8.5 (**c**) scenarios, respectively. The black curve indicates the global evolution considering all countries. Colored curves represent the same evolution aggregating grid cells corresponding to countries being in a similar income class (low, low-middle, high-middle and high). For all groups of countries, the relationship is robustly linear.

Our analysis on labour productivity loss shows the evolution of the relative extra annual loss of total GDP due to the increase of heat exposure in vulnerable sectors, here represented by agriculture, mining and quarrying, manufacturing and construction. The relationship between this productivity loss and CCE is robustly linear at global scale. For each TtC emitted, the annual productivity loss will globally increase by 1.84% (±0.94, 1*σ*-intervals due to climate and inter-model variability), 2.96% (±1.97) and 3.61% (±1.77) of total GDP in the 1% CO_2_, RCP4.5 and RCP8.5 scenarios, respectively (black curves on Fig. 4 and Table 1). This relationship remains approximately unchanged for every additional ton of carbon emitted. In units of constant 2011 international dollars in purchasing power parity (PPP), this represents an annual economic loss that increases with cumulative emissions, reaching a total annual loss of 4,358.73 G$ at cumulative emissions of 1 TtC under the business-as-usual RCP8.5 scenario. This represents an additional annual loss of 0.59 ± 0.26 $ for every ton of CO_2_ (tCO_2_) emitted, while a tCO_2_ is currently purchased at approximately 15 $ on the international market^31^. Today, we may be already experiencing an annual productivity loss due to historical increase in heat exposure of around 1.96% (±0.84) of total GDP at global scale given the RCP8.5 scenario response to total historical emissions (i.e. 555 PgC).

**Table 1:** Annual labour productivity loss ${L}_{\Sigma }$ due to the increase in heat exposure in vulnerable sectors at global scale and classified by income class based on GDP per capita in 2017.

Income Classification	GDP per capita* [US$]	1% CO_2_ [% GDP/TtC]		RCP4.5 [% GDP/TtC]		RCP8.5 [% GDP/TtC]	
		*mean*	*std*	*mean*	*std*	*mean*	*std*
global		1.84	0.94	2.96	1.97	3.61	1.77
low	<995	2.99	1.85	4.37	3.68	5.91	3.60
low-middle	996–3,895	2.89	1.09	5.06	2.57	5.65	2.00
high-middle	3,896–12,055	1.16	0.60	1.96	1.30	2.24	1.10
high	>12,056	0.33	0.20	0.47	0.36	0.66	0.38

The indicator is expressed as a percentage of total GDP per TtC. Mean values (*mean*) and inter-model standard deviations due to climate-induced uncertainties (*std*) are detailed. Low and low-middle income countries exhibit values above average, unlike high and high-middle income countries. *Source: datahelpdesk.worldbank.org.

When data is aggregated by income class according to the GDP per capita of each country, it becomes clear that lower-income countries (according to the World Bank definition^32^) will experience a stronger economic impact compared to higher-income countries (Fig. 4 and Table 1). For instance, the labour productivity loss computed for low- and low-middle-income countries is approximately 9 times higher than the one of high-income countries (red and dark-red curves versus green curves on Fig. 4). These trends deviate significantly from the global average. In contrast, trends for high-middle-income countries (orange lines) are relatively close to the global average. The relationship between changes in the productivity loss and CCE is robustly linear for each country group defined by the four income classes.

**Figure 5:**
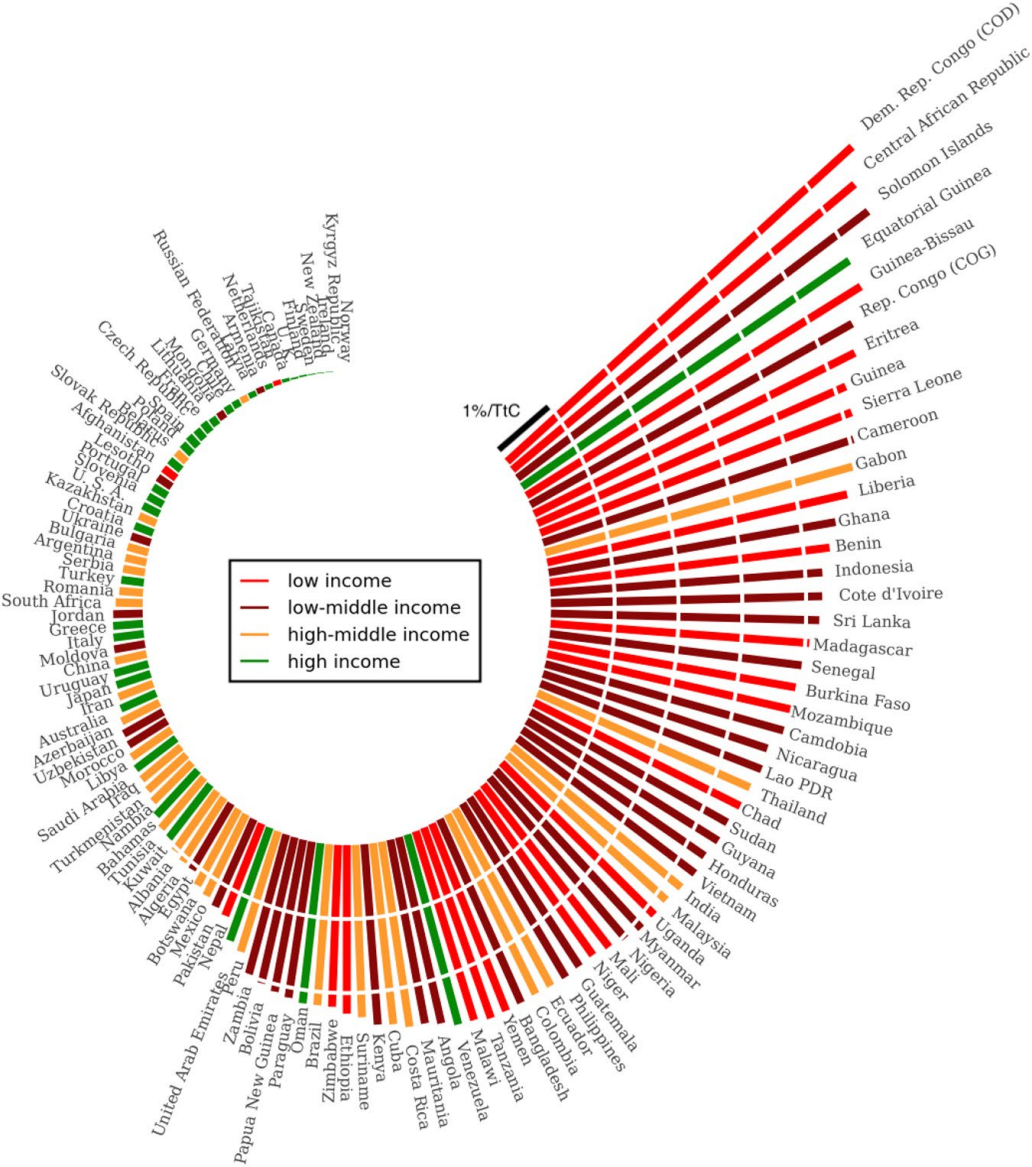
Transient response of annual labour productivity loss ${L}_{\Sigma }$ per carbon emission for each country separately under the 1% CO_2_ scenario. Each bar represents an additional annual loss of 1% of GDP per TtC. One hundred and twenty-seven countries are represented. Other countries are too small to correspond to any model grid cell.

Individual countries experience a wide range of annual labour productivity losses due to the increase in heat exposure according to climate model outputs. They increase with cumulative emissions and range from less than 0.1 to more than 6% of total GDP per TtC emitted, considering only CO_2_ forcing in the 1% CO_2_ scenario (Fig. 5). Again, high-income countries are considerably less affected, with countries such as Canada, Germany, New Zealand and the United Kingdom subject to less than 0.1% of productivity loss per unit emission (consistent with Roson *et al*. (2010)^33^). Note that some individual high-middle income countries are subject to the highest impacts; for example, Gabon, India, Thailand and Malaysia all experience productivity losses from 3 to 5% of total GDP per year for every TtC emitted (consistent with Kjellstrom *et al*. (2013)^34^). These countries may therefore be among the most vulnerable to increasing heat exposure of the labour force, despite their larger financial and institutional resources to adapt compared to the poorest countries. In addition, these countries are also located in regions where several substantial challenges related to climate change can arise simultaneously, such as destructive sea level rise, high rates of population growth and fragile food security^35,36^. However, our analysis clearly demonstrates that lower-income countries will in general be more strongly impacted by the effects of increasing heat exposure compared to higher-income developed countries. When considering non-CO_2_ forcing in the RCP4.5 and RCP8.5 scenarios, impacts on total GDP are greater and the individual ranking of country impacts changes somewhat. However, the overall conclusion remains unchanged (see Supplementary Figs S11 and S12).

## Discussion

In this study, we have estimated a quantitative link between cumulative CO_2_ emissions and potential socioeconomic impacts resulting from changing extreme weather conditions. As such, we are able to quantify the causal relationship between units of CO_2_ that we emit and the effect of increasing heat exposure on labour productivity loss in vulnerable economic sectors based on health recommendations.

In our analysis, we used a simplified version of the WBGT indicator, though our projections of WBGT are similar to those of other studies who have used a more complex version of the indicator [e.g.^37,38^]. Given that temperature and humidity are the main components of the WBGT that change over time with climate change, the assumption of unchanging (average) solar radiation and wind speed does not have a large influence on our results. Furthermore, as the findings of our study are based on the difference over time relative to pre-industrial conditions (rather than on absolute values at particular points in time), the use of a simplified indicator is a reasonable approximation that nevertheless allows us to produce relevant results.

It is worth noting that we have not applied any bias correction to the climate model data that we have used to calculate the WBGT indicators. Though this approach is consistent with other studies that have quantified the response of different climate variables to cumulative emissions [e.g.^9,10,19^], we acknowledge that bias correction is a standard approach for impact studies, and that our study is therefore limited as an assessment of the absolute impacts on labour productivity. However, our primary intent is to calculate a socioeconomic impact per additional emission of CO_2_, and we suggest that this calculation has less sensitivity to bias correction as compared to an estimate of the total impact at a given level of climate change. Furthermore, there are several practical reasons why we consider this to be a reasonable approach. First, corrections to both temperature and humidity data can produce results that are physically inconsistent, and can alter the variability of the modelled time series; as a result, we prefer to use the uncorrected data so as to maintain a clean estimate of modelled change per unit of CO_2_ emissions. In addition, a full bias correction would also require considering model-specific carbon cycle biases that would need to be corrected to account for differences between simulated and observed historical CO_2_ emissions. This is a non-trivial task that has not yet been tackled in the literature.

Another important assumption of our study is that socioeconomic conditions remain static at present-day values. Of course in the real world, socioeconomic conditions continuously evolve over time, and such changes have the potential to either amplify or moderate the economic impacts of climate change. In this sense, our analysis should be interpreted as a “no-adaptation” case, that excludes proactive socioeconomic changes that might decrease the overall impact of increased heat exposure. For example, societies could find ways to adapt to increasing heat exposure by redistributing the workforce within different economic sectors or shifting working hours to seasons or times of day when heat exposure is lower. In a similar manner, we have assumed that the labour output elasticity of all vulnerable sectors is equal to 1, meaning that we assume working hours are lost in direct proportion to elevated heat exposure, and again are not counterbalanced by any short-term adaptation measures [similar to Kjellstrom *et al*. (2009), Hsiang (2010), Wenz and Levermann (2016)^2,39,40^]. There are some previous studies that have considered similar hypotheses to ours, but with evolving socioeconomic conditions^4,41^. These studies estimated an annual loss of 2 to 3% of total GDP at global scale under the RCP8.5 scenario in 2100, which is smaller than our estimates, indicating that there is some potential for adaptation in labour practices to decrease the economic impacts that would be expected without such measures.

A similar caveat applies to the fact that health recommendations are not obligatory and are not always seriously (or consistently) respected at actual work sites. Given this, our estimated loss of GDP is technically what would occur as from worker breaks that are consistent with projected heat exposure, provided that health recommendations are strictly followed [similarly to Takakura *et al*. (2017)^41^].

Another point worth noting is that in our analysis of labour productivity loss, spatial differences of climate conditions and distribution of population are not considered within a country, but are rather averaged for each country. Indeed, statistics of workforce, economic sectors and GDP are only available at a country level^25^. In many cases, this assumption does not have a large bearing on our results: for example, some of the largest countries with uneven population distribution, such as Russia and Canada, are also only marginally affected by productivity loss given their colder climate conditions. Some other large countries such as Brazil and Australia are substantially affected by productivity loss due to the increase in heat exposure, but in these cases the evolution of climate conditions is quite similar over the entire country. The two instances where the effect of within-country population distributions may be important are: (i) countries such as the United States, which span a range of climate changes, have the potential to be sensitive to the distribution of population within the country, though, the changes we find in our study for the United States (Fig. 5) are generally consistent with national estimates found in previous studies^42^, which gives us some confidence that our results are not overly biased by assuming a uniform population and therefore impact distribution within countries; and (ii) we have not considered the distribution of population among urban vs rural areas within a country. It is possible that impacts on productivity loss might be greater in urban areas than what we have estimated based on average national values. However, it is also the case that access to air conditioning is more common in urban workplaces, where the majority of workers do their job indoors.

Similarly to the labour productivity loss that we assessed, the number of productive days lost per year (i.e. the days for which it is supposed to be physically impossible to work) exhibit a clear link to income classes: the lower the income class, the higher the number of days lost (Supplementary Table S4 and Fig. S10). However, the number of days lost computed for low-income countries is (only) 3 to 4 times higher than for high-income countries (compared to a factor of 9 higher for the labour productivity loss), meaning that the fraction of output generated by vulnerable sectors within a country has an influence on our indicator of productivity loss. Indeed, low-income countries generate 73% of their output in vulnerable sectors, unlike higher-income countries (54, 41 and 30%, respectively of the income class)^25^. High-income countries might therefore be better adapted to face the increase in heat exposure on workers^2^.

## Conclusion

The TCRE framework is a robust and highly useful concept, especially when applied to events that are considered as main contributors to the perception of climate change and to mitigation and adaptation responses [e.g.^43^]. It remains a powerful tool in communicating with the general public about how the climate system will respond to further anthropogenic carbon emissions. Our results have extended the generalized TCRE framework from annual and seasonal trends to the increase in heat exposure and its potential socioeconomic impacts. This is the first study to show that the characteristics of heat periods can be robustly linked to cumulative CO_2_ emissions as well as to international climate policy targets. We provide new information that can attribute country-scale climate impacts directly to global cumulative CO_2_ emissions. Using both 1% CO_2_ and RCP scenarios, we have also highlighted that adding non-CO_2_ forcing modifies the amplitude of the transient response of heat exposure, but does not alter its linear relationship with CCE. Furthermore, we have related the loss of labour productivity from increasing heat exposure directly to anthropogenic CO_2_ emissions. We have shown that quantifying the link between CCE and economic and health impacts related to heat exposure is not only possible, but can be an important way to pinpoint impacts at regional scale and incentivize policy-makers to implement appropriate mitigation measures. This work could also be extended to numerous other indicators of extreme weather conditions and related impacts on societies and the economy.

## Methods

### Data and pre-processing

In this study, we used 1% CO_2_ simulations, which consider variations of the CO_2_ concentration only, and historical, RCP4.5 and RCP8.5 simulations (Representative Concentration Pathways), which consider CO_2_ and non-CO_2_ forcing, from eight CMIP5 Earth system models^14,24,44^. The models included in the current ensemble are CanESM2, GFDL-ESM2G, GFDL-ESM2M, HadGEM2-ES, IPSL-CM5A-LR, IPSL-CM5A-MR, IPSL-CM5B-LR and MIROC-ESM (see Supplementary Table S1). This selection corresponds to the ensemble used by Gillett *et al*. (2013)^15^, but we excluded models, for which data necessary to compute heat exposure were not publicly available. For each of these models, a single realization has been taken into account for each simulation. Data considered in this analysis runs from year 1 to 140, from year 1861 to 2005 and from year 2006 to 2095 for the 1% CO_2_, historical and RCP scenarios, respectively. We obtained the evolution of heat exposure from daily data to compute annual statistics. And only then, we interpolated the results onto a common grid for analyses purposes (CanESM2 grid, with a 2.8° spatial resolution), as suggested by Diaconescu *et al*. (2015)^45^ for extreme heat indices. No bias correction has been applied to climate model outputs, similarly to other studies on CCE [e.g.^9,10,19^].

### Definition of heat exposure

Heat exposure is defined by a simplified version of the Wet-Bulb Globe Temperature (WBGT^22,23^). It takes into account variations in temperature and relative humidity of the atmosphere. It is defined as:

1 $${\rm{WBGT}}=0.567\cdot T+0.393\cdot VP+3.94\,[^\circ {\rm{C}}]$$

where *T* is the daily mean temperature in [C], *VP* is the daily vapor pressure in [hPa] and is defined as:

2 $$VP=\frac{q\cdot {P}_{surf}}{0.622+q}[{\rm{Pa}}]$$

where *q* is the specific humidity without dimension and ${P}_{surf}$ the surface pressure in [Pa]. Since the surface pressure is not available as a climate variable in CMIP5 outputs, it is approximated from the sea level pressure ${P}_{sl}$ [Pa], the daily mean temperature *T* [K] and the orography *Z* [m]. This relation has the advantage to be valid for every model configuration^23^:

3 $${P}_{surf}={P}_{sl}\cdot {10}^{\frac{-Z}{18400\cdot T/273.15}}$$

Overall, daily data from ESMs for *T*, *q*, *P*_*sl*_ and *Z* were used to computed our WBGT indicator.

We define the annual total heat exposure ${I}_{\Sigma }$ as the integral of daily WBGT values in a year above a given threshold *θ* considered as the minimum value to consider heat exposure:

4 $${I}_{\Sigma }=\mathop{\int }\limits_{d}\,({{\rm{WBGT}}}_{d}-\theta )\delta d\,[{\rm{K}}\cdot {\rm{days}}]$$

where *d* represents days for which ${\rm{WBGT}}-\theta  > 0$ (see Fig. S4 in the Supporting Information). Differences between ${I}_{\Sigma }$ values at year *t* and at year 0 (i.e. in pre-industrial conditions) are linked to CCE at year *t*.

Six thresholds of heat exposure have been selected from previous studies^1,5,46^ for this analysis: light > 78 °F (25.6 °C); medium > 82 °F (27.8 °C); strong > 85 °F (29.4 °C); very strong > 88 °F (31.1 °C); extreme > 90 °F (32.2 °C); deadly > 95 °F (35 °C) (see Fig. S1 in the Supporting Information).

### Definition of labour productivity loss

Relationships between temperature thresholds mentioned above and health recommendations on labour productivity have been built in previous studies^2–5^. In the framework of our analysis, we assume that health recommendations are followed in every possible context [similarly to Takakura *et al*. (2017)^41^]. A sufficient water intake is considered given the exposure to solar radiation and a mean metabolic rate for human bodies of around 425 W. It means that, during heat exposure considered here as medium, strong, very strong or extreme, workers should rest during 10, 20, 30 or 40 minutes per working hour according to most recent estimations^5^. No recommendations has been published for the threshold considered as deadly^1^, and there is no scientific study recommending to stop working for the entire day^5^. With this information, we can estimate the additional working time lost per year due to the increase in heat exposure in each grid cell *g* by the following indicator:

5 $$\begin{array}{rcl}{\tau }_{g} & = & \frac{1}{6}\cdot \{{N}_{g}({\rm{medium}})-{N}_{g}({\rm{strong}})\}+\frac{1}{3}\cdot \{{N}_{g}({\rm{strong}})-{N}_{g}({\rm{very}}\,{\rm{strong}})\}\end{array}$$

6 $$\begin{array}{lll} &  & +\,\frac{1}{2}\cdot \{{N}_{g}({\rm{very}}\,{\rm{strong}})-{N}_{g}({\rm{extreme}})\}+\frac{2}{3}\cdot {N}_{g}({\rm{extreme}})\end{array}$$

7 $$\begin{array}{rcl} & = & \frac{1}{6}\cdot \{{N}_{g}({\rm{medium}})+{N}_{g}({\rm{strong}})+{N}_{g}({\rm{very}}\,{\rm{strong}})+{N}_{g}({\rm{extreme}})\}\,[{\rm{days}}]\end{array}$$

where *N* is the additional number of days per year above a given temperature threshold. Due to the fact that we use daily mean WBGT values and that working hours are generally warmer than the daily mean, our estimate represents a lower band of the effect of heat exposure on working time loss due to increasing resting times. This indicator is then spatially averaged to get a value of ${\tau }_{c}$ for each country *c* and a value ${\tau }_{\Sigma }$ at global scale.

From statistics of the International Labour Organization^25^, we estimate the change in annual labour productivity (expressed as a relative annual loss of total GDP) for each country *c* and each economic sector *s* considered as vulnerable to heat exposure (agriculture, mining and quarrying, manufacturing and construction). Statistics of economic outputs are considered fixed to values of 2017. In our analysis, we thus assume that all socio-economic conditions remain equal to current ones and that no additional adaptation measures are implemented in the vulnerable sectors selected. The annual loss in labour productivity due to the increase in heat exposure is computed as follows for a specific country:

8 $${L}_{c}=\frac{100}{GD{P}_{c}}\cdot {L}_{c}^{abs}=\frac{100}{GD{P}_{c}}\cdot {\tilde{\tau }}_{c}\cdot \sum _{s}\,{P}_{c,s}[ \% \,{\rm{of}}\,{\rm{GDP}}]$$

where ${\tilde{\tau }}_{c}$ is the mean number of annual working hours lost in the country *c* for an employed person in a vulnerable sector. Moreover, ${p}_{c,s}$ is the mean hourly output of the sector *s*, and $GD{P}_{c}$ is the gross domestic product of the country *c*. Both are expressed in constant 2011 international dollars in PPP.

In more details, ${\tilde{\tau }}_{c}$ and ${P}_{c,s}$ are computed as follows:

9 $${\tilde{\tau }}_{c}=52\cdot {n}_{c}\cdot \frac{{\tau }_{c}}{365}$$

where *n*_*c*_ is the average number of hours worked in a week in country *c*. Vacation time is considered in statistics. For countries with missing values, working hours are assumed to be equal to the global average (i.e. 41.7 hours per week). ${\tau }_{c}$ is divided by 365, since the calendar used in climate simulations does not consider leap years. Days with heat exposure are considered equally distributed among working days, weekends and holidays.

10 $${P}_{c,s}={p}_{c,s}\cdot {E}_{c,s}$$

where ${p}_{c,s}$ is the mean hourly output of an employed person derived from the World Bank database^32^, and ${E}_{c,s}$ is the number of employed persons in the sector *s*. ${E}_{c,s}$ refers to the main activity of the company in which a person worked during the reference period (i.e. 2017), and does not depend on the specific duties of the person’s job, but on the characteristics of the economic sector considered [similarly to Kopp *et al*. (2014)^42^]. Note that to have access to values of ${p}_{c,s}$ and ${E}_{c,s}$ corresponding to categories of activity defined above, modeled estimates database need to be customized on the ILO website under the index named ‘economic activity’.

Finally, the annual loss in labour productivity due to the increase in heat exposure world-wide and aggregated by countries of the same income class can be obtained by the following equation:

11 $${L}_{\Sigma }=\frac{100}{GD{P}_{tot}}\cdot \sum _{c}\,{L}_{c}^{abs}[ \% \,{\rm{of}}\,{\rm{GDP}}]$$

### Multi-model analysis and uncertainty assessment

Simulations of each climate model have been considered equally in the multi-model analysis, without weighting any simulation more than another. Uncertainties are expressed as 1*σ*-intervals of the spread of the 8 models selected. Spatially represented signals (on Fig. 2, for instance) are considered robust under two conditions:

- At least 6 out of 8 climate models agree on the significance of changes.
- The significance of changes in each grid cell shown by one climate model is estimated through a one-tailed Student’s t-test with a 95% confidence interval, considering 20 samples (i.e. years) for both the pre-industrial and future periods.
  For indicators of labour productivity loss, we consider that all socio-economic conditions remain equal to current ones (i.e. 2017). Uncertainties expressed as a relative loss of GDP thus represent 1*σ*-intervals of climate-induced uncertainties only.
  Throughout the manuscript, a relationship between CCE and an indicator of the increase in heat exposure is considered robustly linear, only if the linearity has been verified by the accuracy of a Pearson correlation test:
- Correlation coefficients are above 0.9.
- *p*-values are below the threshold of the 99% confidence interval. The actual value of this limit varies with the number of samples *n* in the distribution. For the 1% CO_2_ scenario, *n* = 140 years × 8 models = 1,120, and the corresponding p-value threshold is 0.0077. For RCP scenarios, *n* = 135 years × 8 models = 1,080, and the corresponding p-value threshold is 0.0078.

## Supplementary information

Supporting Information

## Data Availability

CMIP5 data used in this study is publicly available on all ESGF portals (e.g. https://esgf-node.ipsl.upmc.fr/projects/esgf-ipsl/). Every user has to get an account to access the data in order to prove her/his professional affiliation. GDP data needed for the last part of the study is publicly available as well on the World Bank website (https://data.worldbank.org/indicator/NY.GDP.PCAP.CD). No account is needed to get access. Statistics of the work force in high-risk sectors are also available for free on the International Labour Organization database (https://www.ilo.org/global/statistics-and-databases). Our study has been conducted by analyzing CMIP5 raw data and coding in Python, Matlab and R. After the publication, the code that supports the findings of this study will be available from the corresponding author upon reasonable request.
