# Supplementary material for: Exposure to excessive heat and impacts on labour productivity linked to cumulative CO2 emissions
Source: Sci Rep. 2019 Sep 23;9:13711. doi: 10.1038/s41598-019-50047-w (PMC6757059; doi:10.1038/s41598-019-50047-w)
Supplement: Supplementary file 1 — Supporting Information [file 41598_2019_50047_MOESM1_ESM.pdf]

Supporting Information

Exposure to excessive heat and impacts on labour productivity linked to cumulative CO_2 emissions

Yann Chavaillaz[1,2], Philippe Roy[1], Antti-Ilari Partanen[3], Laurent Da Silva[1], Émilie Bresson[4], Nadine Mengis[5], Diane Chaumont[1] and H. Damon Matthews[2]

[1]Ouranos Inc, 550 rue Sherbrooke Ouest, Tour Ouest 19ème étage, Montréal, QC H3A 1B9, Canada
[2]Department of Geography, Planning and Environment, Concordia University, 1455 boulevard de Maisonneuve Ouest, Montréal, QC H3G 1M8, Canada
[3]Finnish Meteorological Institute, Climate System Research, P.O. Box 503, 00101, Helsinki, Finland
[4]Université du Québec en Abitibi-Témiscamingue, 445 Boulevard de l'Université, Rouyn-Noranda, QC J9X 5E4, Canada
[5]Simon Fraser University, Department of Geography, 8888 University Drive, Burnaby, BC V5A 1S6, Canada

Supplementary Figures

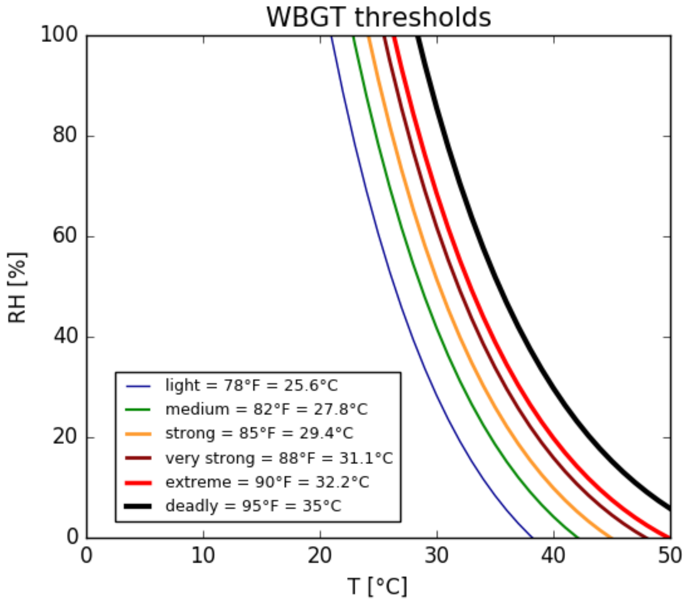

Figure S1: Iso-WBGT of the six WBGT thresholds selected in this study as light, medium, strong, very strong, extreme and deadly. WBGT values depend on both temperature and relative humidity.

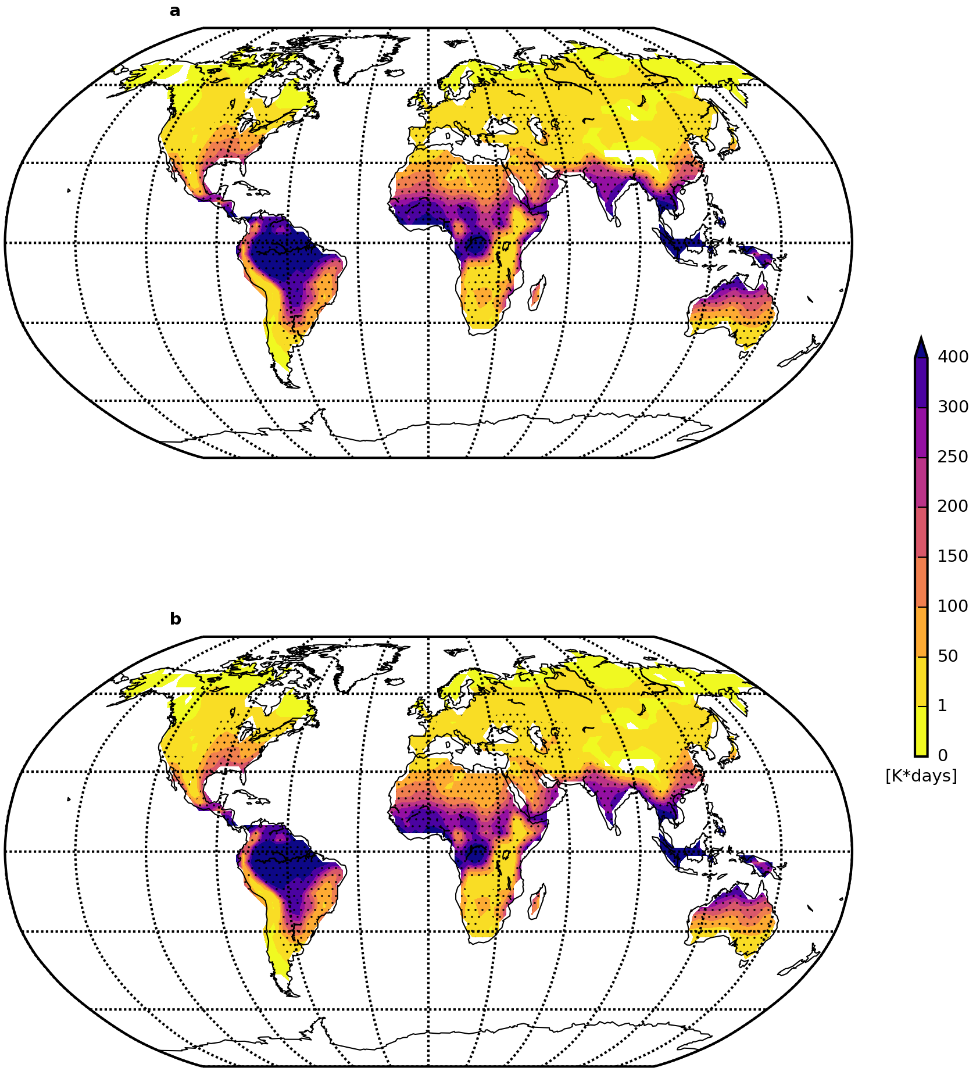

Figure S2: Regional increase in the annual total heat exposure I_Σ with a light WBGT threshold in a 1.5°C-warmer world compared to the pre-industrial period (1861-1880) according to the (**a**) RCP4.5 and (**b**) RCP8.5 scenarios. Dotted regions correspond to where at least 75% of the models agree on the significance of the change.

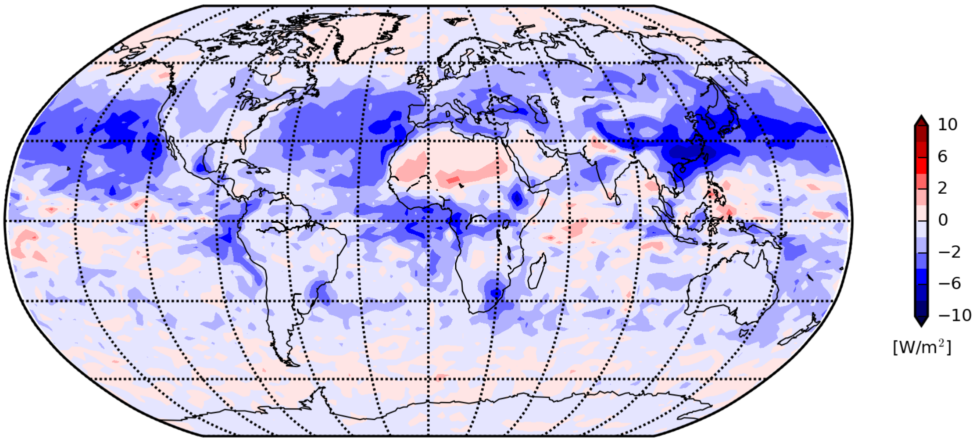

Figure S3: Spatial distribution of aerosol forcing in a 1.5°C-warmer world simulated by the ECHAM-HAMMOZ model (based on Partanen et al. (2018) [46]). Regions with high aerosol forcing correspond to where the 1%CO_2 response is greater than the RCP response (e.g. Chinese coastline). Aerosols thus has an influence in the mitigation of the increase in heat exposure.

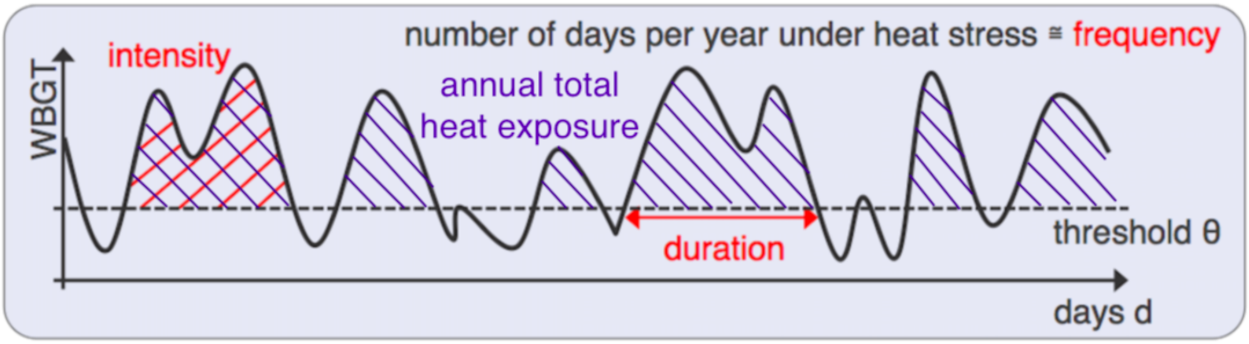

Figure S4: Illustration of the characteristics of heat periods considered in this study. Heat periods can be investigated for their intensity, their duration and their frequency. The annual total heat exposure considers all the episodes and their characteristics to form a one and only indicator. In Figures S5, S6, S7, S8 and S9, we show that the intensity, the duration and the frequency of the annual most severe and the annual average event can also be linked with cumulative carbon emissions.

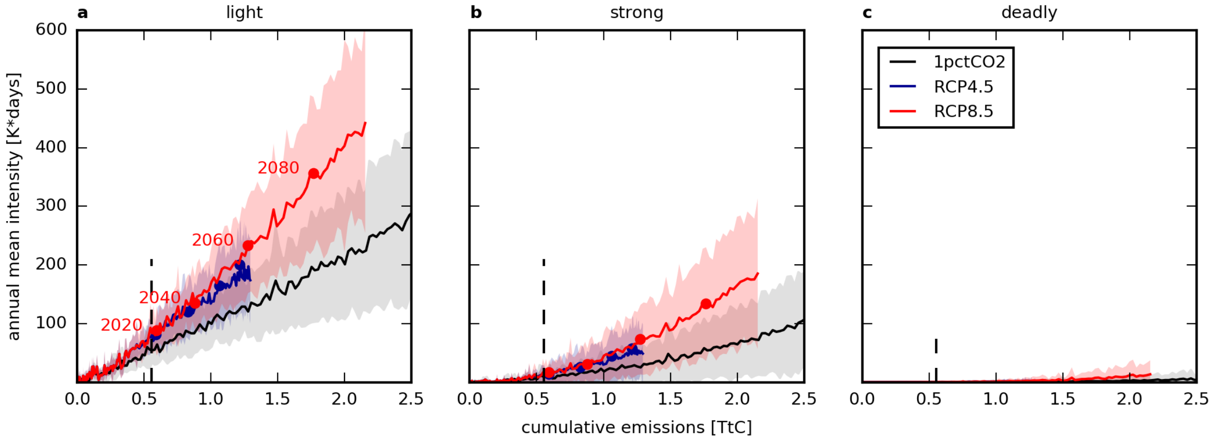

Figure S5: Global increase in the mean intensity of heat periods per carbon emission. Increase in the annual mean intensity of heat periods over land as a function of cumulative carbon emissions for the different scenarios of emissions (1%CO_2, RCP4.5 and RCP8.5) compared to the pre-industrial period (1861-1880). Different WBGT thresholds of heat exposure are considered: light (**a**), strong (**b**) and deadly (**c**). Envelopes correspond to the σ-interval of the inter-model spread.

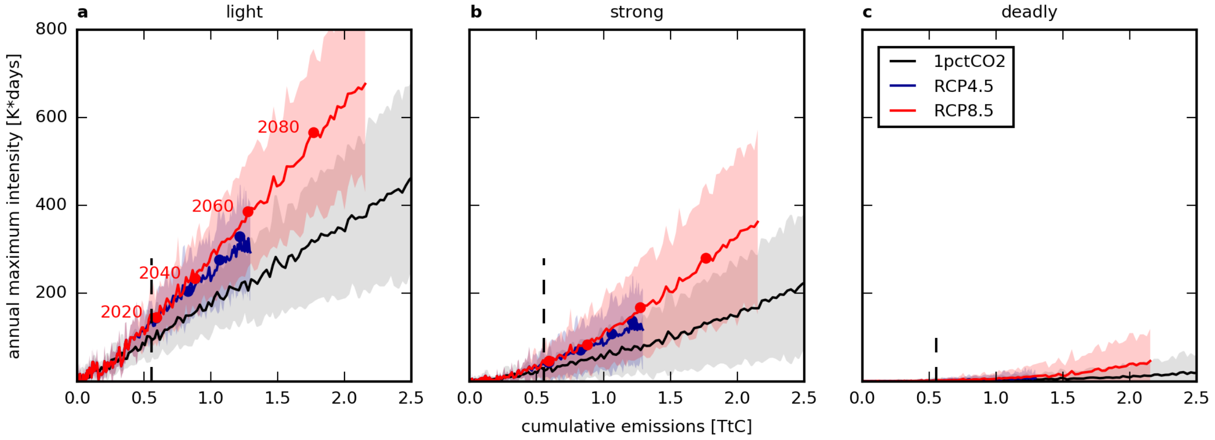

Figure S6: Global increase in the maximum intensity of heat periods per carbon emission. Increase in the annual maximum intensity of heat periods over land as a function of cumulative carbon emissions for the different scenarios of emissions (1%CO_2, RCP4.5 and RCP8.5) compared to the pre-industrial period (1861-1880). Different WBGT thresholds of heat exposure are considered: light (**a**), strong (**b**) and deadly (**c**). Envelopes correspond to the σ-interval of the inter-model spread.

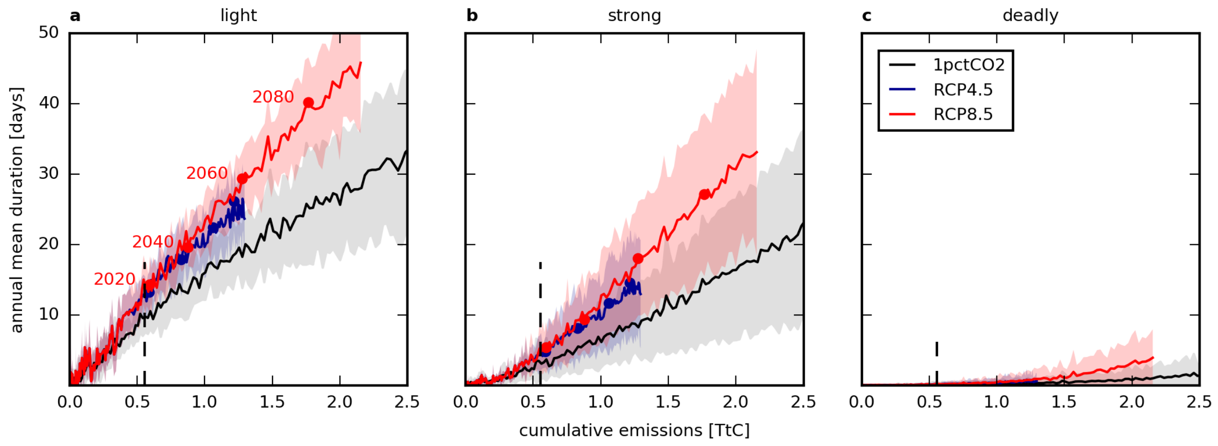

Figure S7: Global increase in the mean duration of heat periods per carbon emission. Increase in the annual mean duration of heat periods over land as a function of cumulative carbon emissions for the different scenarios of emissions (1%CO_2, RCP4.5 and RCP8.5) compared to the pre-industrial period (1861-1880). Different WBGT thresholds of heat exposure are considered: light (**a**), strong (**b**) and deadly (**c**). Envelopes correspond to the σ-interval of the inter-model spread.

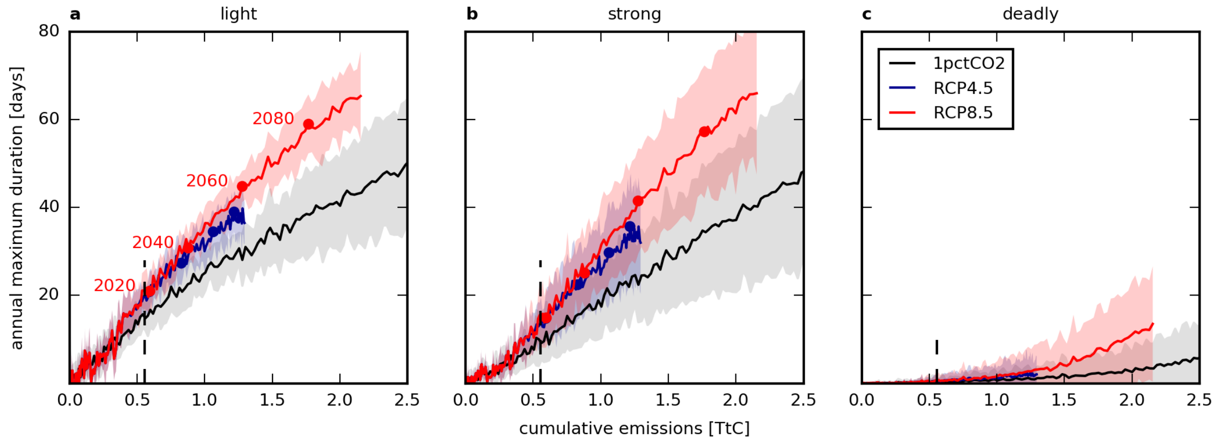

Figure S8: Global increase in the maximum duration of heat periods per carbon emission. Increase in the annual maximum duration of heat periods over land as a function of cumulative carbon emissions for the different scenarios of emissions (1%CO_2, RCP4.5 and RCP8.5) compared to the pre-industrial period (1861-1880). Different WBGT thresholds of heat exposure are considered: light (**a**), strong (**b**) and deadly (**c**). Envelopes correspond to the σ-interval of the inter-model spread.

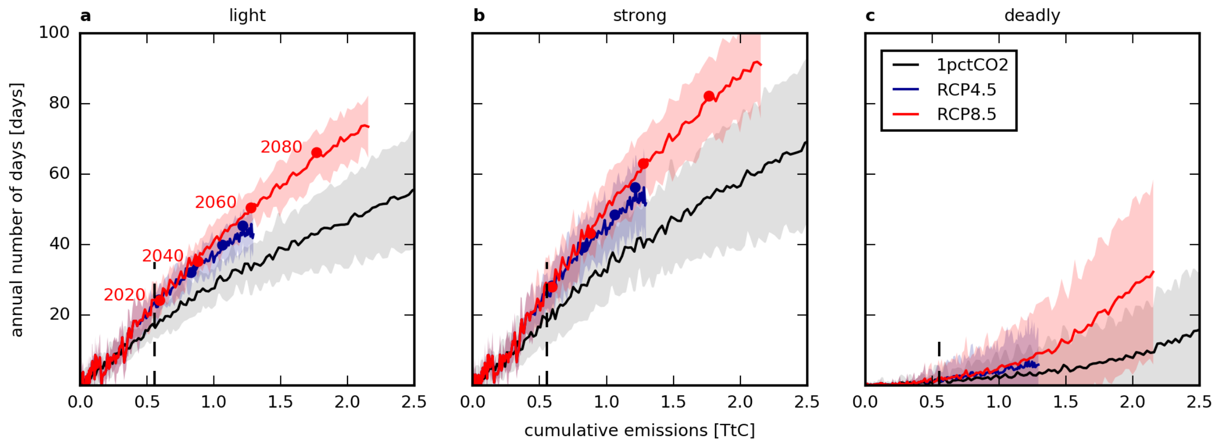

Figure S9: Global increase in the total number of days of heat exposure per carbon emission. Increase in the annual total number of days of heat exposure over land as a function of cumulative carbon emissions for the different scenarios of emissions (1%CO_2, RCP4.5 and RCP8.5) compared to the pre-industrial period (1861-1880). Different WBGT thresholds of heat exposure are considered: light (**a**), strong (**b**) and deadly (**c**). Envelopes correspond to the σ-interval of the inter-model spread.

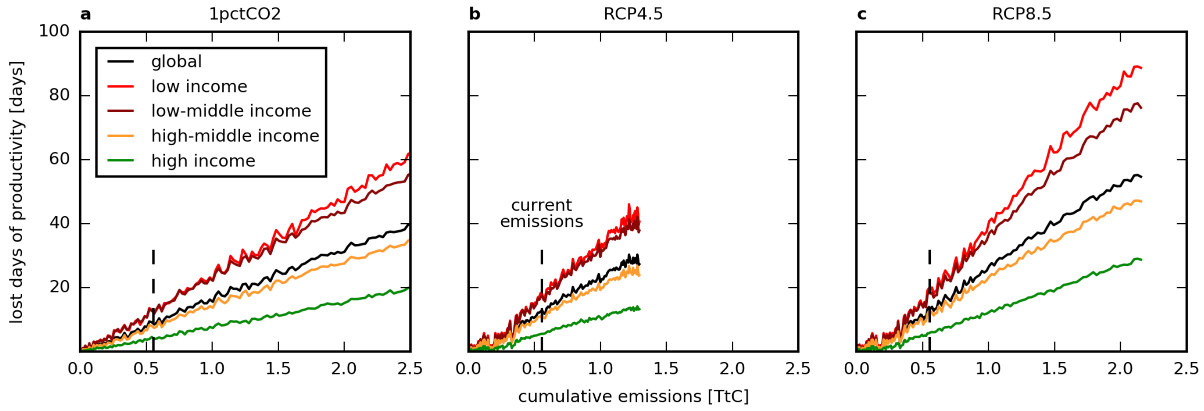

Figure S10: Lost days of productivity τ_Σ due to increase of heat exposure per carbon emission classified by income class based on GDP per capita. The evolution is shown for the (**a**) 1%CO_2, (**b**) RCP4.5 and (**c**) RCP8.5 scenarios, respectively. The black curve indicates the global evolution considering all countries. Colored curves represent the same evolution aggregating grid cells corresponding to countries with a similar income (low, low-middle, high-middle and high). For all groups of countries, the relationship is robustly linear. The lower the income is, the stronger the transient response.

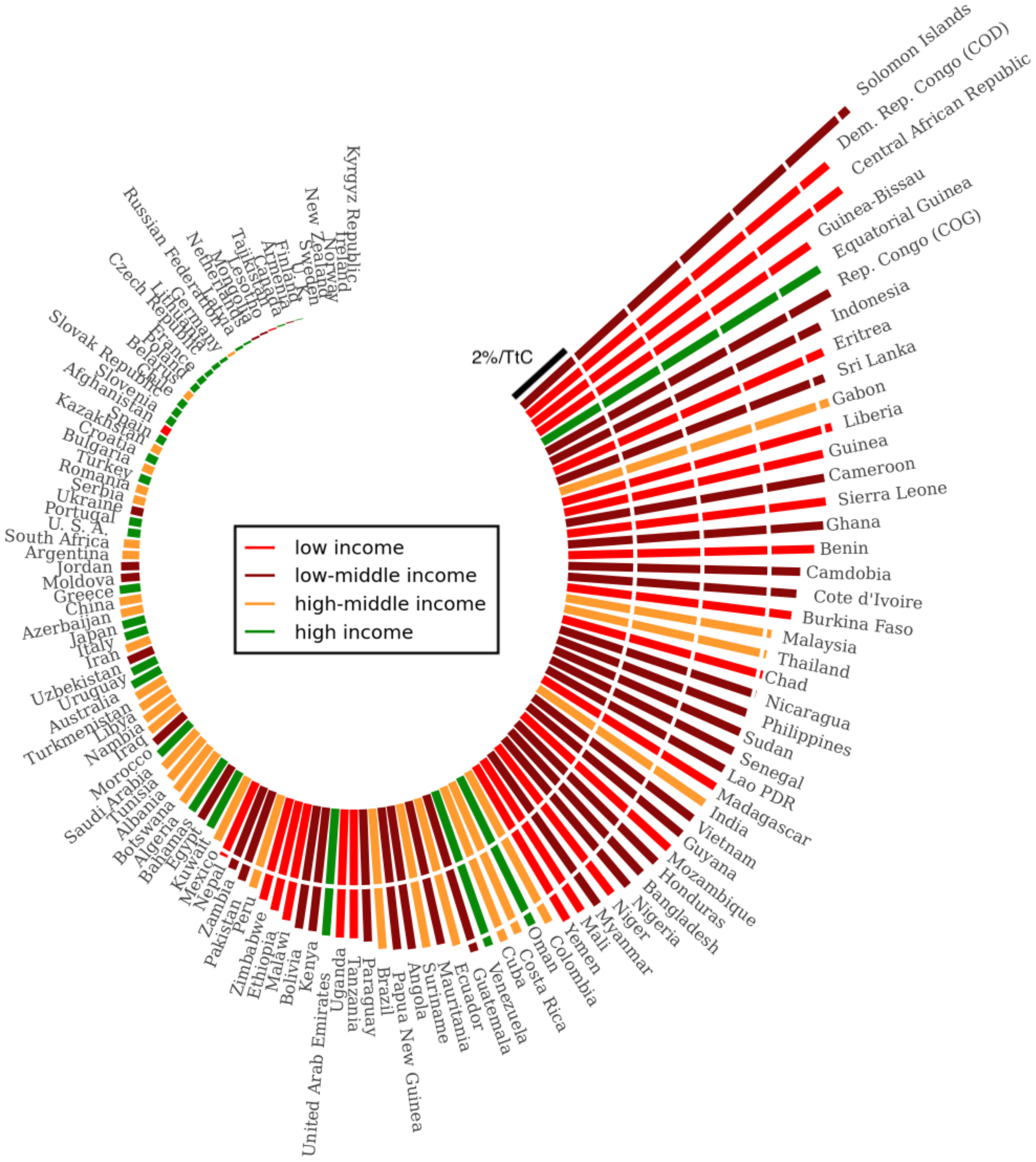

Figure S11: Transient response of annual labour productivity loss L_Σ per carbon emission for each country separately under the RCP4.5 scenario. Each bar represents an additional annual loss of 2% of GDP per TtC. One hundred and twenty-seven countries are represented. Other countries are too small to correspond to any model grid cell.

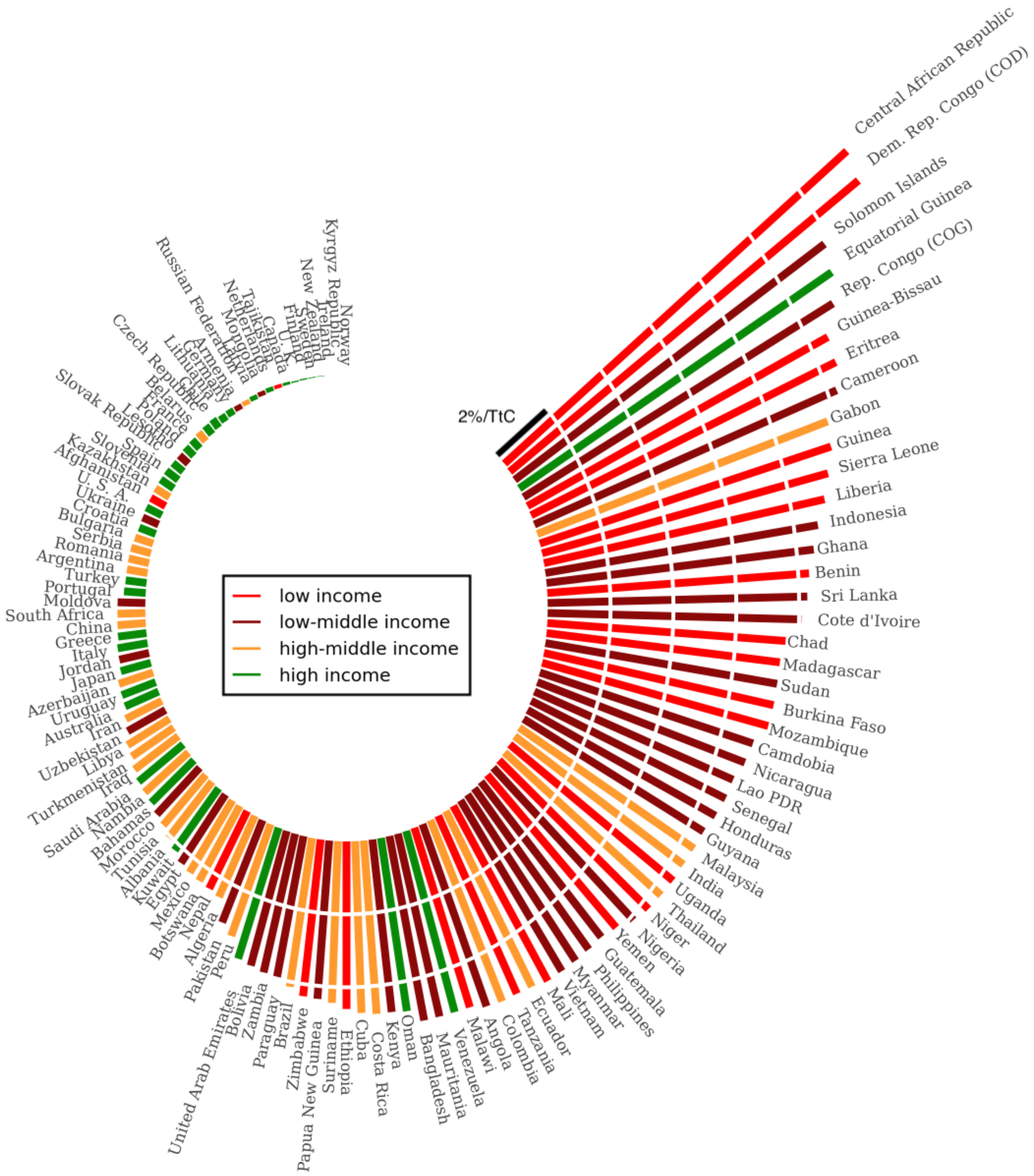

Figure S12: Transient response of annual labour productivity loss L_Σ per carbon emission for each country separately under the RCP8.5 scenario. Each bar represents an additional annual loss of 2% of GDP per TtC. One hundred and twenty-seven countries are represented. Other countries are too small to correspond to any model grid cell.

Supplementary Tables

Table S1: Characteristics of the selected ensemble of Earth System Models.

Model	Modeling Center	Country	Spatial Resolution
CanESM2	CCCma	Canada	2.79x2.81
GFDL-ESM2G	NOAA GFDL	United States	2.02x2
GFDL-ESM2M	NOAA GFDL	United States	2.02x2.5
HadGEM2-ES	MOHC	United Kingdom	1.25x1.88
IPSL-CM5A-LR	IPSL	France	1.89x3.75
IPSL-CM5A-MR	IPSL	France	1.27x2.5
IPSL-CM5B-LR	IPSL	France	1.89x3.75
MIROC-ESM	MIROC	Japan	2.79x2.81

Table S2: Regions selected from the IPCC SREX report [28].

Short name	Long name	Subregions
ECNA	Eastern and Central North America	CNA, 4<br>ENA, 5
IND	India	SAS, 23
AUS	Australia	NAU, 25<br>SAU, 26
NSA	Northern South America	AMZ, 7<br>NEB, 8
NCA	Northern Central Africa	SAH, 14<br>WAF, 15<br>EAF, 16
SEA	South-East Asia	SEA, 24

Table S3: Transient Response of the annual total heat exposure I_Σ, expressed in [K·days/TtC] for SREX regions. Mean values and inter-model standard deviations are detailed.

**region**	**threshold**	**1%CO_2** [K·days/TtC]		**RCP4.5** [K·days/TtC]		**RCP8.5** [K·days/TtC]	
		mean	*std*	*mean*	*std*	*mean*	*std*
global	*light*	213.1	105.1	282.2	90.70	360.0	110.6
	medium	164.1	92.49	202.3	84.24	278.8	105.3
	strong	121.1	80.35	133.4	76.94	208.6	99.13
	very strong	78.31	64.72	69.14	62.35	136.9	87.69
	extreme	55.30	53.31	40.55	50.34	97.07	77.25
	deadly	18.44	28.37	10.53	23.33	31.98	46.12
ECNA	*light*	190.6	102.6	240.7	91.72	324.2	120.4
	medium	142.3	85.80	165.4	79.97	242.8	110.8
	strong	101.6	71.23	106.3	69.18	177.4	100.4
	very strong	62.56	56.31	51.73	53.83	113.1	86.47
	extreme	42.94	45.86	29.26	41.77	78.78	75.38
	deadly	13.64	22.77	8.07	17.78	25.53	44.05
IND	*light*	389.3	178.4	558.0	149.2	660.8	167.8
	medium	325.4	165.7	440.41	148.7	555.4	172.1
	strong	255.2	149.6	311.8	142.3	441.4	173.0
	very strong	175.2	124.4	177.6	119.1	308.3	162.5
	extreme	127.6	103.4	112.7	97.02	227.2	146.1
	deadly	44.48	52.58	29.78	44.67	81.71	88.53
AUS	*light*	199.0	102.4	270.6	96.09	334.1	113.8
	medium	155.3	88.90	200.0	83.35	260.2	103.6
	strong	120.0	76.71	144.4	72.99	202.2	95.08
	very strong	81.78	62.87	83.87	60.07	140.1	82.64
	extreme	58.79	53.43	49.71	49.96	102.0	72.95
	deadly	19.25	27.38	9.19	19.45	32.46	42.71
NSA	*light*	539.1	240.4	762.1	234.6	895.9	235.6
	medium	448.9	241.3	589.2	261.4	749.7	262.5
	strong	343.3	233.6	396.0	274.8	581.3	282.6
	very strong	229.9	204.4	208.5	240.0	391.1	276.1
	extreme	167.9	175.1	126.5	196.0	283.1	253.3
	deadly	59.67	98.87	34.40	82.98	101.4	155.9
NCA	*light*	539.1	240.4	762.1	234.6	895.9	235.6
	medium	448.9	241.3	589.2	261.4	749.7	262.5
	strong	343.3	233.6	396.0	274.8	581.3	282.6
	very strong	229.9	204.4	208.4	240.0	391.1	276.1
	extreme	167.9	175.1	126.5	196.0	283.1	253.3
	deadly	59.67	98.87	34.40	82.98	101.4	155.9
SEA	*light*	519.2	207.2	802.2	165.8	913.8	168.5
	medium	477.6	201.1	711.9	162.7	845.4	173.1
	strong	397.2	185.8	536.9	155.0	715.8	175.1
	very strong	261.6	157.4	268.0	126.0	496.8	167.7
	extreme	173.6	123.7	125.0	85.24	346.3	148.3
	deadly	32.08	30.44	6.14	8.89	73.95	62.36

Table S4: Lost days of productivity τ_Σ at global scale classified by country groups based on GDP per capita in 2017, expressed in [days/TtC]. Mean values (*mean*) and inter-model standard deviations (*std*) are detailed. Low and low-middle income countries exhibit values above average, unlike high and high-middle income countries. *Source: `datahelpdesk.worldbank.org`.

**Income Classification**	**GDP per capita*** [US$]	**1%CO_2** [days/TtC]		**RCP4.5** [days/TtC]		**RCP8.5** [days/TtC]	
		mean	*std*	*mean*	*std*	*mean*	*std*
global		15.5	6.6	22.4	6.1	26.1	5.1
low	<995	24.6	10.9	33.2	9.8	42.0	9.6
low-middle	996 - 3,895	21.7	9.2	31.0	7.8	36.6	6.8
high-middle	3,896 - 12,055	13.5	5.9	19.7	5.6	22.5	4.6
high	>12,056	8.0	4.2	10.5	3.9	13.5	4.0
